# The Role of Extracellular Vesicles in Demyelination of the Central Nervous System

**DOI:** 10.3390/ijms21239111

**Published:** 2020-11-30

**Authors:** José Antonio López-Guerrero, Inés Ripa, Sabina Andreu, Raquel Bello-Morales

**Affiliations:** 1 Departamento de Biología Molecular, Universidad Autónoma de Madrid, Cantoblanco, 28049 Madrid, Spain; ja.lopez@uam.es (J.A.L.-G.); ines.ripa@cbm.csic.es (I.R.); sandreu@cbm.csic.es (S.A.); 2 Centro de Biología Molecular Severo Ochoa, CSIC-UAM, Cantoblanco, 28049 Madrid, Spain

**Keywords:** extracellular vesicles, demyelination, central nervous system, multiple sclerosis, neuromyelitis optica, progressive multifocal leukoencephalopathy, herpesviruses

## Abstract

It is being increasingly demonstrated that extracellular vesicles (EVs) are deeply involved in the physiology of the central nervous system (CNS). Processes such as synaptic activity, neuron-glia communication, myelination and immune response are modulated by EVs. Likewise, these vesicles may participate in many pathological processes, both as triggers of disease or, on the contrary, as mechanisms of repair. EVs play relevant roles in neurodegenerative disorders such as Alzheimer’s or Parkinson’s diseases, in viral infections of the CNS and in demyelinating pathologies such as multiple sclerosis (MS). This review describes the involvement of these membrane vesicles in major demyelinating diseases, including MS, neuromyelitis optica, progressive multifocal leukoencephalopathy and demyelination associated to herpesviruses.

## 1. Introduction

Extracellular vesicles (EVs) are a heterogeneous group of double-layered phospholipid membrane vesicles secreted by most cell types belonging to the three domains of life, Bacteria, Archaea and Eukarya [1,2,3,4]. EVs have been isolated from numerous biological fluids such as blood, amniotic fluid, saliva, cerebrospinal fluid (CSF), ascitic fluid and urine [5,6,7]. EVs are currently considered to be key mediators of intercellular communication, and are increasingly being associated with physiological and pathological processes across all fields of biomedicine, including cancer [8,9], immune response [10,11,12,13] and infection [14]. EVs may enclose proteins, lipids, nucleic acids, metabolites or even pathogens, and their participation in viral infection has been extensively demonstrated, both for enveloped and naked viruses [15,16,17]. EVs influence viral entry, spread and immune evasion, and these vesicles may play crucial roles in communication between infected and uninfected cells [18,19].

The classification and nomenclature of EVs is challenging, given the current difficulty of separating complex populations of EVs into subtypes of particular size, composition and biogenesis pathway [4]. However, three major categories of EVs can be broadly established: apoptotic bodies, microvesicles (MVs) and exosomes, distinguishable by their size, markers, biogenesis, release pathways and function [20]. MVs derive from shedding of the plasma membrane [21,22], and they have a heterogeneous size, ranging from 100 nm to 1 μm in diameter [23]. These shedding vesicles are enriched in lipid rafts and proteins such as flotillin-1 or integrins [24], and expose phosphatidylserine (PS) on the outer leaflet of the plasma membrane [25]. Exosomes are the intraluminal vesicles released to the extracellular space after fusion of multivesicular bodies (MVBs) with the cell membrane [26]. They have a typical diameter of 30–100 nm and are enriched in tetraspanins such as CD9, CD63 and CD81, and endosomal markers including TSG101 and Alix [27]. Exosome biogenesis is regulated by endosomal sorting complexes required for transport ESCRT machinery and, therefore, ESCRT proteins and the accessory proteins Alix, TSG101, HSC70 or HSP90β are generally found in exosomes. However, exosomes release may also depend on sphingomyelinase, an ESCRT-independent mechanism.

However, although tetraspanins were initially considered specific markers of exosomes, these proteins have also been found in MVs and apoptotic bodies [20]. The presence of cytosolic and cell membrane-associated proteins in MVs is easily understandable, given that these vesicles are formed by budding of the plasma membrane [20].

Several methods to isolate EVs are currently available. The classical method separates EVs by ultracentrifugation. The relative centrifugal force needed to isolate MVs varies frequently between 10,000 and 20,000× *g* [28], whereas around 100,000× *g* are typically used to pellet exosomes [13,29,30,31]. To avoid the co-precipitation of unwanted structures such as apoptotic bodies or protein aggregates, density gradient centrifugation may be a useful alternative to differential centrifugation [32]. Filtration can also be used alone or in combination with these methods. EVs can also be isolated, targeting their different protein markers by immune-magnetic beads. Other methods to isolate EVs include microfluidic devices, size exclusion chromatography and precipitation protocols using polymers [33].

EVs can enter recipient cells by fusion with the plasma membrane or by endocytic pathways. Endocytosis can be dependent or independent of clathrin, and may also involve other mechanisms, such as phagocytosis, macropinocytosis, caveolin-dependent uptake or lipid raft-mediated endocytosis [34].

The multifaceted functions of EVs in the nervous system are increasingly being studied [35,36,37,38], revealing that EVs are crucial mediators of processes such as inflammation [39,40], myelination [41,42] and neuron-glia communication [43,44]. In addition, several studies have reported the participation of EVs in myelination and demyelination [45]. In this review, we will describe the role of EVs in diseases of the central nervous system (CNS), focusing on current knowledge about the involvement of EVs in demyelinating processes.

## 2. Extracellular Vesicles in the CNS

Several studies have evidenced the significant contribution of EVs to physiological and pathological processes of the CNS, with bi-directional effects: providing protection against injury or favoring disease. EVs are secreted by all neural cell types [35,36,46]. These vesicles have a functional role in several physiological processes such as development, myelination, regeneration, immune response or regulation of synaptic activity, and might be involved in neuropathology or to the contrary, in neuro-regeneration and repair [35,36,47]. EVs have been implicated in brain tumors [48,49,50,51,52], stroke [53,54,55] and neurodegenerative disorders such as Alzheimer’s or Parkinson’s diseases [56,57,58,59,60], viral infections of the nervous system [61] and demyelinating pathologies such as multiple sclerosis (MS) [62,63,64]. In neurodegeneration, EVs have been proposed as vehicles for the packaging and spread of toxic or misfolded proteins [65,66,67]. For instance, phosphorylated tau protein has been found in secreted exosomes [68]. A fraction of beta-amyloid peptide is secreted into the extracellular medium associated with exosomes, and exosomal proteins are also enriched in the amyloid plaques [69]. Regarding Parkinson’s disease, α-synuclein—a neuronal protein whose misfolding and aggregation are linked to pathology—is exported via exosomes [70]. The enzyme Cu/Zn superoxide dismutase 1 (SOD1), whose alteration is associated with amyotrophic lateral sclerosis, is also associated with exosomes [71,72]. Furthermore, exosomes may also contribute to the spread of prions, since cellular prion protein (PrPc) and the abnormally folded PrP scrapie (PrPsc) have been associated with exosomes, which were additionally shown to be infectious [73].

EVs have also been implicated in demyelinating diseases. The myelin sheath is a lipid-rich layer that electrically insulates axons and allows the saltatory conduction of action potentials. Myelin is a crucial evolutionary advancement that permits an increased speed of nerve impulses without a concomitant rise in axon diameter. Regarding EVs, oligodendrocytes (OLs), the myelin-forming cells of the CNS, also secrete both exosomes and MVs, which may influence myelination and neuron-glia communication [43,74,75]. OLs secrete exosomes harboring several enzymes and myelin proteins such as myelin basic protein (MBP), 2′3′-cyclic nucleotide phosphodiesterase (CNPase), myelin oligodendrocyte glycoprotein (MOG) and proteolipid protein (PLP), the major myelin protein in the CNS [43,76]. Moreover, in response to neuronal signals, OLs secrete exosomes in an electric activity-dependent manner which are internalized by neurons, affecting their activity. Conversely, neurons can also secrete exosomes that may influence myelin maintenance and regeneration [41]. In addition, OLs stimulated with glutamate secrete exosomes which, when internalized by neurons, have been shown to exert several positive effects, such as resistance to oxidative stress (via transfer of superoxide dismutase and catalase), enhancement of neuronal survival during oxygen-glucose deprivation and increased neuronal firing rate [43,77]. Therefore, exosomes secreted by OLs after neuronal signals may transport components for myelin membrane biogenesis and, moreover, they may transfer trophic and survival factors to nearby axons under homeostatic and stress conditions, in order to support myelination and myelin maintenance [41].

## 3. Demyelinating Diseases of the CNS

Demyelinating diseases of the CNS are acquired pathologies characterized by a primary destruction of central myelin sheaths, although myelin damage may concomitantly induce damage to axons [78,79]. Conversely, axon injury can also trigger secondary destruction of myelin [80]. Demyelinating diseases must not be confused with dysmyelinating diseases or leukodystrophies, in which myelin damage is produced by a genetically determined process [81,82]. Several causes, such as inflammation and viral infections, may lead to demyelination, although those triggers may also interact to produce disease [83].

**Figure 1:**
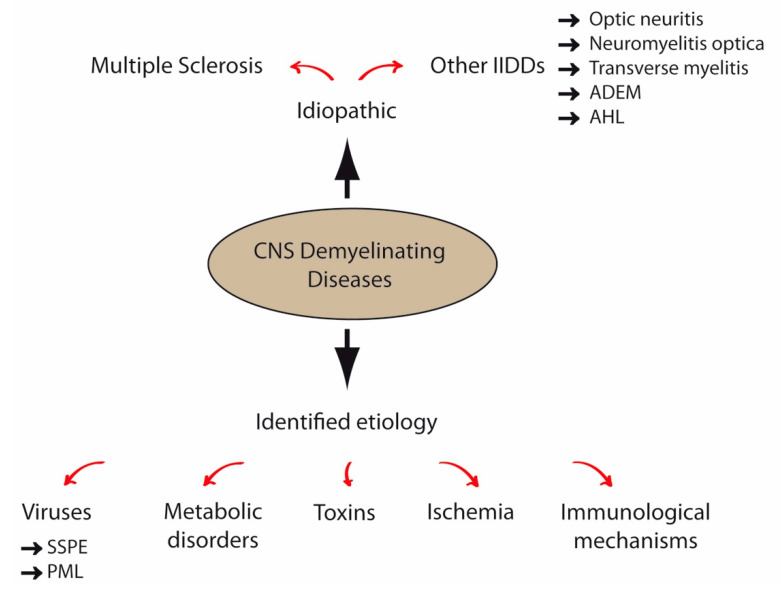
Major demyelinating diseases of the central nervous system (CNS). The major demyelinating inflammatory CNS disorder is multiple sclerosis (MS), a progressive autoimmune disease of unknown etiology. There are other idiopathic inflammatory-demyelinating diseases (IIDDs) such as optic neuritis, neuromyelitis optica and transverse myelitis. Another IIDD is acute disseminated encephalomyelitis (ADEM), an inflammatory autoimmune disorder with a likely infectious etiology. Acute hemorrhagic leukoencephalitis (AHL), a variant of ADEM, is possibly elicited by an infectious trigger. Other demyelinating CNS diseases have an identified etiology, such as viral infections, for instance, subacute sclerosing panencephalitis (SSPE) or progressive multifocal leukoencephalopathy (PML), immunological mechanisms, toxins, metabolic disorders or ischemia.

The major demyelinating inflammatory disorder is MS, a progressive autoimmune disease of unknown etiology characterized by inflammation, blood–brain barrier (BBB) disruption, demyelination, OL and axonal loss and gliosis [84]. In addition, there are other idiopathic inflammatory-demyelinating diseases (IIDDs) such as optic neuritis, neuromyelitis optica (NMO) and transverse myelitis [85]. Acute disseminated encephalomyelitis (ADEM) is also an inflammatory autoimmune disorder of the CNS myelin, but, in this case, an infectious etiology has been observed in most cases: the first symptoms are frequently preceded by viral or bacterial infections [79]. Acute hemorrhagic leukoencephalitis (AHL), a variant of ADEM, is a human autoimmune disorder probably caused by molecular mimicry stimulated by viral or bacterial pathogens [86]. On the other hand, several demyelinating CNS diseases have a defined etiology such as viral infection, immunological mechanisms, toxins, metabolic disorders or ischemia (Figure 1).

Several species have been linked to viral demyelination (Figure 1). For example, progressive multifocal leukoencephalopathy (PML) is a rare human demyelinating disease caused by the JC virus (JCV). Subacute sclerosing panencephalitis (SSPE) has also been related to viral infection in humans. Thus, several years after infection, persistent measles virus (MeV) may trigger destruction of OLs and neurons in a progressive course that presents a high mortality rate [81]. Although vaccination has reduced the number of cases of measles (and therefore, SSPE), recent anti-vaccination campaigns have resulted in a worrying increase in measles outbreaks [87]. In addition, this disease remains endemic in many developing countries [88].

In animals, the neurotropic coronavirus mouse hepatitis virus (MHV) may also induce neurovirulence and demyelination, depending on the strain and route of inoculation [89]. In particular, strains JHM and A59 can infect the brain and induce demyelination. Ten days after intracranial inoculation, the A59 strain is usually cleared, but mice can develop demyelination at 3 to 4 weeks post-infection [89]. Regarding the JHM strain, the virus is not cleared from the CNS, and consequently it can produce persistent infection [90]. The JHM strain spike (S) glycoprotein has been reported to be the major determinant of neurovirulence [91].

## 4. Role of EVs in Demyelinating Diseases of the CNS

### 4.1. Multiple Sclerosis

MS is an immune-mediated, demyelinating disease of the CNS of unknown etiology. The disease is multifactorial, and it is probably influenced by a complex array of interactions between genes and the environment [84,92,93]. The pathology of MS is characterized by multifocal demyelinating lesions in both the white and gray matter of the brain and spinal cord [84,94], and lesions are probably caused by infiltration of immune cells into the CNS, where auto-reactive lymphocytes attack myelin antigens [95]. These lesions can be associated with axonal degeneration and chronic neurodegeneration. MS usually starts with a single episode of neurological dysfunction, the “clinically isolated syndrome” (CIS), which sometimes can evolve towards a definite MS diagnosis. MS is usually multiphasic (relapsing-remitting MS), with reversible episodes of neurological symptoms (relapses). However, occasionally it may present as a progressive course (primary progressive MS) [84]. In another form of the disease (secondary progressive MS), patients undergo the relapsing-remitting form prior to a progressive accumulation of neurological damage [96]. Although the etiology of MS is still unknown, several viruses have been proposed to be involved in its pathogenesis [97,98,99].

One of the earliest events during the development of MS is a compromise of the BBB. In healthy individuals, brain-endothelial tight junctions limit adhesion and migration of immune cells into the CNS. However, inflammation can increase expression of adhesion molecules such as intracellular adhesion molecule 1 (ICAM-1), vascular cell adhesion molecule 1 (VCAM-1), E-selectin, or platelet-endothelial cell adhesion molecule-1 (PECAM-1) [100], which allow leukocytes to cross the BBB. Adhesion of activated leukocytes to brain endothelial cells and the subsequent transendothelial migration through the impaired BBB are considered major events in the pathogenesis of MS. Moreover, activated leukocytes secrete pro-inflammatory cytokines, such as interleukin-1 beta (IL-1β), tumor necrosis factor alpha (TNFα) and interferon gamma (IFNγ), which can disrupt the BBB and increase its permeability. Pro-inflammatory cytokines also enhance leukocyte endothelial adhesion and migration and stimulate shedding of endothelial MVs [101]. Pro-inflammatory cytokines released by effector T cells, mostly Th1 and Th17, are key players in BBB alteration, via the increase of adhesion molecules on endothelial cells that enables lymphocytes adhesion and infiltration into the CNS [102]. BBB disruption can be detected as a leakage of gadolinium chelates—contrast agents used in magnetic resonance imaging—into the CNS [103,104]. BBB impairment has been suggested as an essential step in demyelination seen in MS, though it is still unclear whether BBB rupture is causative or rather a consequence of MS [100].

A possible role for endothelial to mesenchymal transition (EndoMT)—a process by which endothelial cells lose their specialized function and de-differentiate into mesenchymal cells—during BBB dysfunction in MS pathogenesis has also been suggested [100]. In fact, it has been demonstrated that transforming growth factor beta 1 (TGF-β1) and IL-1β drive EndoMT in a human brain endothelial cell line and, in addition, vascular alterations associated with EndoMT in MS brain lesions have been observed, suggesting that EndoMT might underlie BBB dysfunction during MS pathophysiology [105].

After demyelination, a natural mechanism aiming to repair the damaged myelin starts. This process is mediated by the generation of new mature OLs derived from OL precursor cells (OPCs), which are recruited to the lesions. However, remyelination can often decline due to multiple reasons, including the unavailability of OPCs and OLs in lesions, a lack of myelin sheath assembly despite OPC and mature OL availability, the presence of a hostile environment in lesions, or an altered expression of regulatory molecules [106,107].

**Figure 2:**
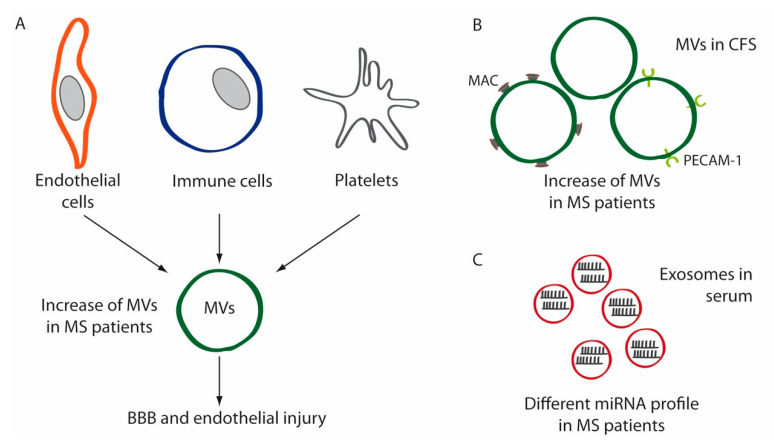
Schematic diagrams depicting the implication of extracellular vesicles (EVs) in MS. (**A**) MVs secreted by endothelial cells, immune cells and platelets are increased in the plasma of MS patients compared to healthy controls. These MVs contribute to blood–brain barrier (BBB) disruption and endothelial injury in these patients. (**B**) MVs isolated from the cerebrospinal fluid (CSF) of MS patients are increased compared to controls. These EVs are enriched in membrane attack complex (MAC) components and platelet-endothelial cell adhesion molecule-1 (PECAM-1). (**C**) Circulating exosomes have a different miRNA profile in MS patients.

Although our understanding is still incomplete, different studies have demonstrated that EVs, both exosomes and MVs, may be involved in MS pathogenesis [108] (Figure 2A). MVs released from different cell types, including endothelial cells, astrocytes, leukocytes, platelets and myeloid cells, have been linked to MS lesions [62]. A therapeutic potential for EVs in demyelinating diseases has also been suggested [63]. In this regard, several studies have shown the therapeutical potential of EVs from different sources [109,110,111]. It has been reported that EVs secreted from OLs may stimulate remyelination in the CNS. Pusic et al. showed the ability of exosomes to improve myelination via OPCs differentiation into mature myelin-producing cells, suggesting that exosomes might be a useful therapy for remyelination [42]. On the other hand, EVs may also be neuroprotective after brain injury [112]. Regarding inflammation, the therapeutic effects of exosomes loaded with curcumin or a Stat3 inhibitor have been demonstrated in lipopolysaccharide (LPS)-induced brain inflammation and myelin oligodendrocyte glycoprotein-induced experimental autoimmune encephalomyelitis mouse models [113], suggesting that this therapeutic approach might be useful for brain inflammatory diseases. Exosomes transporting RNAs and proteins have also shown a therapeutic potential. Small interfering RNA delivery mediated by exosomes demonstrated a strong knockdown of a therapeutic target in mice [114].

The first suggestion of a role for EVs in MS was reported by Scolding et al. in 1989 [115], when they observed an increase of EVs enriched in membrane attack complex (MAC) components in CSF isolated from MS patients, and its augmented shedding from the surface of cultured OLs (Figure 2B). The authors suggested that those EVs might be participating in recovery from cell injury via the shedding of vesicles enriched in MACs [115]. Later, Minagar et al. [116] measured the release of MVs into plasma in patients with MS (both in exacerbation and in remission) compared to healthy controls. Results showed that MVs expressing PECAM-1 were significantly increased in patients with MS during exacerbation but not during remission (Figure 2B), suggesting endothelial dysfunction [116]. In addition, plasma from patients with MS was able to induce endothelial cell dysfunction in vitro [116]. Subsequent studies revealed that injured endothelium released MVs that bound to monocytes and activated them, and this binding led to an enhanced inflammation and facilitated transendothelial migration. In addition, circulating MV–monocyte complexes were significantly increased in MS patients during exacerbations compared to remissions, a finding that was corroborated by magnetic resonance imaging [117]. Endothelial MVs from MS patients were also shown to enhance monocyte migration through the endothelium in vivo, an effect that was inhibited by IFN-β 1b [118]. The increased serum levels of markers such as PECAM-1 in MS patients, as well as the increase in PECAM-1-positive MVs, are signals of the endothelial and BBB dysfunction in MS [119,120,121,122].

Subsequent reports analyzing the content of myeloid MVs in the CSF of MS and CIS patients also revealed higher levels of EVs compared to controls [123]. A later study confirmed the increase of endothelial and platelet MVs in plasma from MS patients compared to healthy controls [124]. In addition, to analyze the effect of MVs isolated from MS patients on endothelial barrier function, human umbilical vein endothelial cells HUVEC cultures were incubated with MVs from MS patients and healthy donors, and the response of endothelial barrier to MVs was measured by electric cell-substrate impedance sensing. Results showed that MVs from MS patients induced a stronger disruption of the endothelial barrier than controls [124].

Among their diverse cargo, exosomes can transport functional RNAs, mostly mRNAs and micro-RNAs (miRNAs), which can stimulate different phenotypic responses in recipient cells [125]. In this sense, the differential expression of exosomal miRNAs in MS patients’ sera [126,127] (Figure 2C) has been recently reported, showing that circulating exosomes have a different RNA profile that may distinguish relapsing-remitting from progressive disease and suggesting that exosomal-associated miRNAs may be useful biomarkers for MS. Given the ability of Th17 cells to release large amounts of the pro-inflammatory cytokine IL-17, and the role of Treg cells promoting tolerance, the balance between these two lymphocytes subsets plays a central role in autoimmunity. Cellular miRNAs have been proposed as mechanisms of inflammation involved in the pathogenesis of MS, altering the balance of Th17/Treg cells [128,129,130]. Specifically, miR-155, which expression is upregulated in MS lesions [131], has been shown to promote the development of inflammatory Th1/Th17 cell subsets [132]. Enhanced secretion of EVs containing miRNA-155 has been suggested as a mechanism of inflammation in MS [102,129].

### 4.2. Neuromyelitis Optica (NMO)

**Figure 3:**
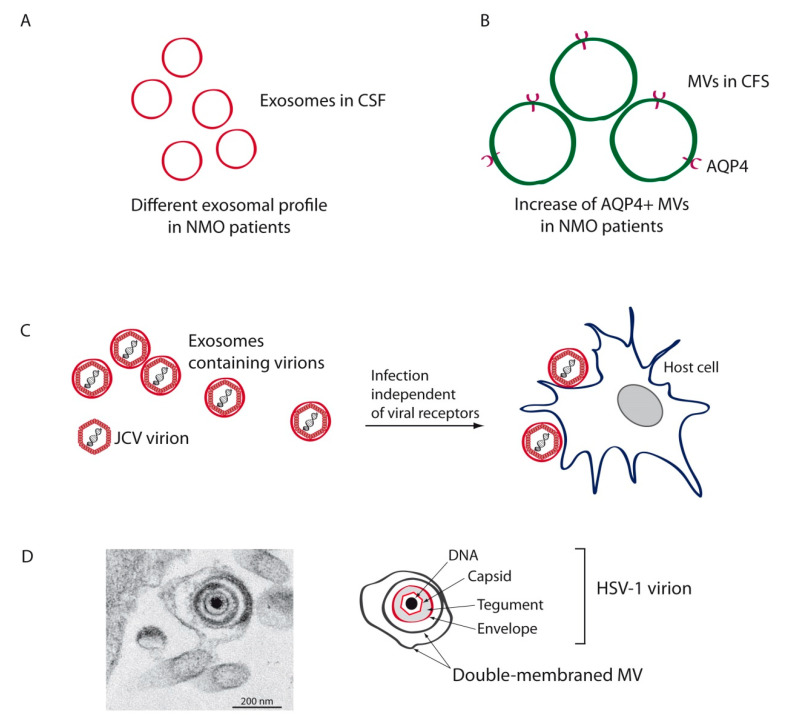
Schematic diagrams depicting the implication of EVs in other CNS demyelinating diseases. Analysis of the CSF of neuromyelitis optica (NMO) patients has shown a distinct exosomal profile (**A**) and an increase in astrocytic aquaporin-4 (AQP4)-positive microvesicles (MVs) (**B**) in these patients compared to healthy controls. (**C**) JCV, the virus associated with the etiology of progressive multifocal leukoencephalopathy (PML), may be secreted enclosed in exosomes that can infect target cells independently of viral receptors. (**D**) Herpes simplex virus type 1 (HSV-1), another virus implicated in demyelination, may also be spread enclosed in MVs. Transmission electron micrograph shows a HSV-1 virion enclosed in a double-membraned MV, and is thus covered by three lipid membrane layers.

NMO is an autoimmune inflammatory disease of the CNS in which pathogenic antibodies auto-react against the astrocytic aquaporin-4 (AQP4) water channel protein, affecting the optic nerve and spinal cord [133]. This IIDD was initially considered a variant of MS, but the discovery of autoantibodies against AQP4 led to its recognition as a distinct disease. Exosomes isolated from the CSF of NMO patients have displayed specific variations compared to those of MS and idiopathic longitudinally extensive transverse myelitis (Figure 3A), indicating that CSF exosomes may be a useful means to differentiate those diseases [134]. An increase of AQP4-positive EVs has recently been reported in the CSF of an NMO patient (Figure 3B), suggesting that these EVs might influence the pathogenesis and could serve as biomarkers for this disease [135]. Further research will be necessary to ascertain whether AQP4 autoantibodies found in NMO are triggered by an increase in AQP4-positive EVs.

### 4.3. Progressive Multifocal Leukoencephalopathy

PML is a rare disease of the CNS caused by JCV [136], named for John Cunningham, the first patient from whom the virus was isolated in 1971 [137]. JCV, a nonenveloped double-stranded DNA virus that belongs to the *Polyomaviridae* family [138], is a widespread opportunistic human pathogen which infects more than 70% of the population. JCV infection is asymptomatic and frequently occurs early in life, establishing latency in several cell types and organs such as the brain, tonsils, lungs, B lymphocytes and kidneys [139]. Under certain poorly understood circumstances, usually accompanied by host immunosuppression, the virus may spread to the CNS, crossing the BBB and reactivating to cause PML. Thus, latent JCV, termed the “archetype” form that is incapable of productively infecting glial cells [140], may transform into the neurotropic “prototype” form, which may in turn infect OLs and cause CNS disease [139]. Within the CNS, JCV infects OLs and astrocytes and induces demyelination. PML involves progressive damage to white matter elicited by the infection and subsequent loss of OLs, resulting in demyelination and neurodegeneration [140]. PML can be a serious complication in MS and AIDS patients, and it has been frequently associated with other autoimmune and inflammatory diseases such as rheumatoid arthritis or systemic lupus erythematosus. It can also be a side effect of treatment such as monoclonal antibody therapy or immunosuppressant drugs [136].

In immunocompetent individuals, JCV attachment is mediated by pentasaccharide lactoseries tetrasaccharide c (LSTc) [141]. The serotonin receptor 5-hydroxytryptamine (5-HT) mediates viral entry [142], which proceeds via clathrin-mediated endocytosis. Since OLs and astrocytes lack LSTc receptors, additional models of transmission have been hypothesized and examined, finding that JCV may spread by EVs [138,143,144]. In this regard, recent investigations carried out with the SVG-A cell line found JCV particles enclosed in EVs which were able to infect target cells independently of viral receptors (Figure 3C). Treatment with neutralizing antisera was only able to reduce infectivity of purified virions, whereas treatment of EV-associated virus had no effect [143]. In addition, choroid plexus epithelial cells infected with JCV were also demonstrated to produce virion-containing EVs. These EVs, which expressed exosomal markers such as CD9 and TSG101, entered glial cells by macropinocytosis and clathrin-dependent endocytosis [144]. This mode of infection by EVs probably plays a crucial role in JCV’s spread into and within the CNS, since the major targets of JCV (OLs and astrocytes) do not express the viral receptors needed for viral entry.

### 4.4. EVs as Putative Means for Herpesvirus Spread with Relevance in Demyelination

There is no single microorganism that has been accepted to be the causal agent of MS, and indeed, several pathogens have been associated with this disease, including bacteria—such as *Chlamydia pneumoniae* and *Staphylococcus aureus*—and viruses, especially members of the family *Herpesviridae* [98,99,145,146] and endogenous retroviruses (ERV) [147]. However, it may be more likely that viruses act not as causative agents but rather as risk factors. Some herpesviruses have been implicated in demyelinating diseases, especially Epstein-Barr virus (EBV) [93,148,149] and human herpesvirus 6 (HHV6) [150,151]. In addition, studies in murine models and human patients have suggested a link between herpes simplex virus type 1 (HSV-1) and the infectious etiology of demyelinating diseases [147], although the ubiquitous nature of this virus makes it difficult to study the role of this herpesvirus in demyelination. A direct epidemiological association between microbial agents and MS is difficult for several reasons: (1) the agent in question may be cleared by the time of diagnosis, (2) specific antibodies or autoreactive T cells may be present both in patients and healthy controls and (3) microorganisms may inhibit immune responses at later stages [152].

Several systematic reviews and meta-analyses have studied the involvement of HHV6 in MS, and although some of them have shown a significant relationship [150], others concluded that further work is necessary to corroborate the link [153]. On the other hand, HHV6 type A has been detected predominantly in MS lesions, and initial studies based on detection of viral DNA in the CSF supported a pathogenic role for HHV6 in MS, although later studies showed contradictory results [152].

Regarding the gammaherpesvirus EBV, several findings have also suggested its association with MS [149,154]. Several epidemiological studies have shown that acquiring infectious mononucleosis later in life is a risk factor for MS. Also, viral DNA in the blood has been detected more frequently in MS patients compared to healthy controls [152]. EBV may also contribute to MS pathogenesis by activating human ERV-W [154]. In summary, the causative role of EBV in MS remains controversial.

The presence of oligoclonal IgG bands (OCBs) in the CSF of patients is a hallmark of MS. OCBs are characteristically detected in inflammatory and infectious disorders of the CNS (they are found in the CSF of greater than 95% of MS patients), and they are an indication of an anomalous intrathecal B-cell response [147]. OCBs directed against EBV and HHV6 [155] as well as HSV-1 [156] have been identified in MS patients, although other studies reported opposing results [157], with the antigen reactivity of most OCBs remaining unidentified, to date.

All subfamilies belonging to the *Herpesviridae* are known to exploit EVs during their viral cycle [158]. In this regard, the betaherpesvirus HHV6 can modify the molecular transport machinery in infected cells, and the exosome secretion pathway plays a significant role in its life cycle. Indeed, HHV6 virions are released via the exosomal pathway and, similarly, the human gammaherpesvirus EBV may exploit exosomes to enhance viral infection [158]. Participation of the alphaherpesvirus HSV-1 in secretion of EVs is widely accepted. In fact, the transfer of exosomes secreted by cells infected with HSV-1 containing viral RNAs and interferon stimulator genes (STING) to uninfected cells was discovered not long ago [159,160]. Infected cells appear to use exosomes as a mechanism to transport STING, along with mRNAs, miRNAs and the CD9 exosome marker to uninfected cells, suggesting that HSV-1 may control infection spread and limit its virulence in order to facilitate spread among individuals. Moreover, our recent studies have shown that MVs released by HSV-1-infected cells may harbor viral particles, which can be endocytosed by naïve cells leading to productive infection (Figure 3D). This mechanism can allow HSV-1 to expand its tropism and evade the immune response [16,161]. However, in spite of the evidence suggesting involvement of some herpesviruses in demyelination, and despite the capacity of those viruses to exploit the EV pathway, no data has been reported that confirms that EVs secreted by infected cells serve as causative factors for demyelinating diseases.

### 4.5. Human Endogenous Retroviruses

There is a solid epidemiological association between MS and the expression of human ERVs, which is upregulated in the brains of MS patients compared to healthy controls [162,163,164,165,166]. In addition, the MS-associated retrovirus (MSRV) has been frequently isolated from MS patients, and its expression in the CSF has been linked with the rate of progression of the disease [167,168]. Also involved in MS, syncytins are Env glycoproteins encoded by ERV genes that stimulate cell-to-cell fusion in mammalian placental morphogenesis, promoting the formation of syncytia [169,170]. In this regard, syncytin-1, which can trigger neuroimmune activation and OL damage [171], may also inhibit the differentiation of oligodendroglial precursors, a process that can lead to remyelination failure [172]. In addition, expression of syncytin-1 in glial cells is upregulated in demyelinating lesions of MS patients. In this context, HSV-1 has been demonstrated to upregulate syncytin-1 [173], and therefore, a role for this virus in HERV-mediated demyelination should not be discarded. However, an association of HERV with MS etiology has not been demonstrated, although immunopathogenic properties of syncytin-1 suggest an influence on MS clinical manifestations [152]. Horizontal gene transfer mediated by EVs carrying syncytin-1 has been demonstrated, suggesting that endogenous syncytin-1 facilitates fusion of EVs with target cells [174]. Moreover, syncytins incorporated in exosomes have been demonstrated to mediate cell uptake [175]. Nevertheless, regarding the pathogenesis of MS, no studies have yet linked EVs to the spread of HERV.

## 5. Conclusions

EVs are crucially involved in the physiology and pathology of the CNS. Physiological processes such as neuron-glia communication or myelination and disorders such as Alzheimer’s or Parkinson’s diseases, viral infections and demyelinating diseases, are significantly affected by EVs. MVs secreted by endothelial or immune cells are increased in plasma from MS patients compared to healthy controls. These MVs may contribute to BBB disruption and endothelial injury in these patients. In addition, circulating exosomes have a different miRNA profile in MS patients, a fact that may be useful to distinguish relapsing-remitting from progressive disease, and suggests that exosomal-associated miRNAs may be useful biomarkers in MS. EVs isolated from CSF and serum of MS and NMO patients are also increased compared to controls and, finally, HSV-1, another virus associated to demyelination, may also be spread enclosed in MVs, a mechanism that can permit HSV-1 to expand its tropism and evade the immune response. Overall, these findings demonstrate that it is essential to deepen the knowledge of EVs, since these vesicles may be not only markers, but also therapeutic targets of demyelination. Drug development and therapeutic treatments for demyelinating diseases may, in the future, depend on the knowledge of EVs. This review has described the function of EVs in CNS diseases, focusing on MS, neuromyelitis optica, progressive multifocal leukoencephalopathy and demyelination associated to herpesviruses. Further investigation will be necessary to determine the involvement of EVs in other demyelinating diseases of the CNS.

## References

[1] Yanez-Mo M., Siljander P.R., Andreu Z., Zavec A.B., Borras F.E., Buzas E.I., Buzas K., Casal E., Cappello F., Carvalho J. (2015). Biological properties of extracellular vesicles and their physiological functions. J. Extracell. Vesicles.

[2] Van Niel G., D’Angelo G., Raposo G. (2018). Shedding light on the cell biology of extracellular vesicles. Nat. Rev. Mol. Cell Biol..

[3] Gyorgy B., Szabo T.G., Pasztoi M., Pal Z., Misjak P., Aradi B., Laszlo V., Pallinger E., Pap E., Kittel A. (2011). Membrane vesicles, current state-of-the-art: Emerging role of extracellular vesicles. Cell. Mol. Life Sci. Cmls.

[4] Margolis L., Sadovsky Y. (2019). The biology of extracellular vesicles: The known unknowns. PLoS Biol..

[5] Kalra H., Drummen G.P., Mathivanan S. (2016). Focus on Extracellular Vesicles: Introducing the Next Small Big Thing. Int. J. Mol. Sci..

[6] Zaborowski M.P., Balaj L., Breakefield X.O., Lai C.P. (2015). Extracellular Vesicles: Composition, Biological Relevance, and Methods of Study. Bioscience.

[7] Yuana Y., Sturk A., Nieuwland R. (2013). Extracellular vesicles in physiological and pathological conditions. Blood Rev..

[8] Jurj A., Pop-Bica C., Slaby O., Stefan C.D., William C.C., Korban S.S., Berindan-Neagoe I. (2020). Tiny Actors in the Big Cellular World: Extracellular Vesicles Playing Critical Roles in Cancer. Int. J. Mol. Sci..

[9] Barros F.M., Carneiro F., Machado J.C., Melo S.A. (2018). Exosomes and Immune Response in Cancer: Friends or Foes?. Front. Immunol..

[10] Veerman R.E., Gucluler Akpinar G., Eldh M., Gabrielsson S. (2019). Immune Cell-Derived Extracellular Vesicles—Functions and Therapeutic Applications. Trends Mol. Med..

[11] Robbins P.D., Dorronsoro A., Booker C.N. (2016). Regulation of chronic inflammatory and immune processes by extracellular vesicles. J. Clin. Investig..

[12] Tian J., Casella G., Zhang Y., Rostami A., Li X. (2020). Potential roles of extracellular vesicles in the pathophysiology, diagnosis, and treatment of autoimmune diseases. Int. J. Biol. Sci..

[13] Thery C., Ostrowski M., Segura E. (2009). Membrane vesicles as conveyors of immune responses. Nat. Rev. Immunol..

[14] Silverman J.M., Reiner N.E. (2011). Exosomes and other microvesicles in infection biology: Organelles with unanticipated phenotypes. Cell. Microbiol..

[15] Schorey J.S., Cheng Y., Singh P.P., Smith V.L. (2015). Exosomes and other extracellular vesicles in host-pathogen interactions. Embo Rep..

[16] Bello-Morales R., Lopez-Guerrero J.A. (2018). Extracellular Vesicles in Herpes Viral Spread and Immune Evasion. Front. Microbiol..

[17] McNamara R.P., Dittmer D.P. (2020). Extracellular vesicles in virus infection and pathogenesis. Curr. Opin. Virol..

[18] Raab-Traub N., Dittmer D.P. (2017). Viral effects on the content and function of extracellular vesicles. Nat. Rev. Microbiol..

[19] Meckes D.G. (2015). Exosomal communication goes viral. J. Virol..

[20] Doyle L.M., Wang M.Z. (2019). Overview of Extracellular Vesicles, Their Origin, Composition, Purpose, and Methods for Exosome Isolation and Analysis. Cells.

[21] Cocucci E., Meldolesi J. (2015). Ectosomes and exosomes: Shedding the confusion between extracellular vesicles. Trends Cell Biol..

[22] Cocucci E., Racchetti G., Meldolesi J. (2009). Shedding microvesicles: Artefacts no more. Trends Cell Biol..

[23] Raposo G., Stoorvogel W. (2013). Extracellular vesicles: Exosomes, microvesicles, and friends. J. Cell Biol..

[24] Del Conde I., Shrimpton C.N., Thiagarajan P., Lopez J.A. (2005). Tissue-factor-bearing microvesicles arise from lipid rafts and fuse with activated platelets to initiate coagulation. Blood.

[25] Wei X., Liu C., Wang H., Wang L., Xiao F., Guo Z., Zhang H. (2016). Surface Phosphatidylserine Is Responsible for the Internalization on Microvesicles Derived from Hypoxia-Induced Human Bone Marrow Mesenchymal Stem Cells into Human Endothelial Cells. PLoS ONE.

[26] Colombo M., Raposo G., Thery C. (2014). Biogenesis, secretion, and intercellular interactions of exosomes and other extracellular vesicles. Annu. Rev. Cell Dev. Biol..

[27] Andreu Z., Yanez-Mo M. (2014). Tetraspanins in extracellular vesicle formation and function. Front. Immunol..

[28] Gould S.J., Raposo G. (2013). As we wait: Coping with an imperfect nomenclature for extracellular vesicles. J. Extracell. Vesicles.

[29] Witwer K.W., Buzas E.I., Bemis L.T., Bora A., Lasser C., Lotvall J., Nolte-’t Hoen E.N., Piper M.G., Sivaraman S., Skog J. (2013). Standardization of sample collection, isolation and analysis methods in extracellular vesicle research. J. Extracell. Vesicles.

[30] Bello-Morales R., Lopez-Guerrero J.A. (2020). Isolation/Analysis of Extracellular Microvesicles from HSV-1-Infected Cells. Methods Mol. Biol..

[31] Lane R.E., Korbie D., Trau M., Hill M.M. (2017). Purification Protocols for Extracellular Vesicles. Methods Mol. Biol..

[32] Momen-Heravi F., Balaj L., Alian S., Mantel P.Y., Halleck A.E., Trachtenberg A.J., Soria C.E., Oquin S., Bonebreak C.M., Saracoglu E. (2013). Current methods for the isolation of extracellular vesicles. Biol. Chem..

[33] Konoshenko M.Y., Lekchnov E.A., Vlassov A.V., Laktionov P.P. (2018). Isolation of Extracellular Vesicles: General Methodologies and Latest Trends. Biomed Res. Int..

[34] Mulcahy L.A., Pink R.C., Carter D.R. (2014). Routes and mechanisms of extracellular vesicle uptake. J. Extracell. Vesicles.

[35] Lai C.P., Breakefield X.O. (2012). Role of exosomes/microvesicles in the nervous system and use in emerging therapies. Front. Physiol..

[36] Kramer-Albers E.M., Hill A.F. (2016). Extracellular vesicles: Interneural shuttles of complex messages. Curr. Opin. Neurobiol..

[37] Basso M., Bonetto V. (2016). Extracellular Vesicles and a Novel Form of Communication in the Brain. Front. Neurosci..

[38] Holm M.M., Kaiser J., Schwab M.E. (2018). Extracellular Vesicles: Multimodal Envoys in Neural Maintenance and Repair. Trends Neurosci..

[39] Horstman L.L., Jy W., Ahn Y.S., Minagar A., Alexander J.S. (2017). Cell-Derived Microparticles/Exosomes in Neuroinflammation. Inflammatory Disorders of the Nervous System: Pathogenesis, Immunology, and Clinical Management.

[40] Pascual M., Ibanez F., Guerri C. (2020). Exosomes as mediators of neuron-glia communication in neuroinflammation. Neural Regen. Res..

[41] Domingues H.S., Falcao A.M., Mendes-Pinto I., Salgado A.J., Teixeira F.G. (2020). Exosome Circuitry During (De)(Re)Myelination of the Central Nervous System. Front. Cell Dev. Biol..

[42] Pusic K.M., Pusic A.D., Kraig R.P. (2016). Environmental Enrichment Stimulates Immune Cell Secretion of Exosomes that Promote CNS Myelination and May Regulate Inflammation. Cell. Mol. Neurobiol..

[43] Fruhbeis C., Frohlich D., Kuo W.P., Amphornrat J., Thilemann S., Saab A.S., Kirchhoff F., Mobius W., Goebbels S., Nave K.A. (2013). Neurotransmitter-triggered transfer of exosomes mediates oligodendrocyte-neuron communication. PLoS Biol..

[44] Fields R.D., Stevens-Graham B. (2002). New insights into neuron-glia communication. Science.

[45] Selmaj I., Mycko M.P., Raine C.S., Selmaj K.W. (2017). The role of exosomes in CNS inflammation and their involvement in multiple sclerosis. J. Neuroimmunol..

[46] Colombo E., Borgiani B., Verderio C., Furlan R. (2012). Microvesicles: Novel biomarkers for neurological disorders. Front. Physiol..

[47] Budnik V., Ruiz-Canada C., Wendler F. (2016). Extracellular vesicles round off communication in the nervous system. Nat. Rev. Neurosci..

[48] Ciregia F., Urbani A., Palmisano G. (2017). Extracellular Vesicles in Brain Tumors and Neurodegenerative Diseases. Front. Mol. Neurosci..

[49] Kucharzewska P., Christianson H.C., Welch J.E., Svensson K.J., Fredlund E., Ringner M., Morgelin M., Bourseau-Guilmain E., Bengzon J., Belting M. (2013). Exosomes reflect the hypoxic status of glioma cells and mediate hypoxia-dependent activation of vascular cells during tumor development. Proc. Natl. Acad. Sci. USA.

[50] Graner M.W., Cumming R.I., Bigner D.D. (2007). The heat shock response and chaperones/heat shock proteins in brain tumors: Surface expression, release, and possible immune consequences. J. Neurosci. Off. J. Soc. Neurosci..

[51] Graner M.W., Alzate O., Dechkovskaia A.M., Keene J.D., Sampson J.H., Mitchell D.A., Bigner D.D. (2009). Proteomic and immunologic analyses of brain tumor exosomes. FASEB J. Off. Publ. Fed. Am. Soc. Exp. Biol..

[52] Mallawaaratchy D.M., Hallal S., Russell B., Ly L., Ebrahimkhani S., Wei H., Christopherson R.I., Buckland M.E., Kaufman K.L. (2017). Comprehensive proteome profiling of glioblastoma-derived extracellular vesicles identifies markers for more aggressive disease. J. Neuro-Oncol..

[53] Liu W., Bai X., Zhang A., Huang J., Xu S., Zhang J. (2019). Role of Exosomes in Central Nervous System Diseases. Front. Mol. Neurosci..

[54] Doeppner T.R., Herz J., Gorgens A., Schlechter J., Ludwig A.K., Radtke S., de Miroschedji K., Horn P.A., Giebel B., Hermann D.M. (2015). Extracellular Vesicles Improve Post-Stroke Neuroregeneration and Prevent Postischemic Immunosuppression. Stem Cells Transl. Med..

[55] Otero-Ortega L., Laso-Garcia F., Gomez-de Frutos M.D., Rodriguez-Frutos B., Pascual-Guerra J., Fuentes B., Diez-Tejedor E., Gutierrez-Fernandez M. (2017). White Matter Repair After Extracellular Vesicles Administration in an Experimental Animal Model of Subcortical Stroke. Sci. Rep..

[56] Soria F.N., Pampliega O., Bourdenx M., Meissner W.G., Bezard E., Dehay B. (2017). Exosomes, an Unmasked Culprit in Neurodegenerative Diseases. Front. Neurosci..

[57] Mathews P.M., Levy E. (2019). Exosome Production Is Key to Neuronal Endosomal Pathway Integrity in Neurodegenerative Diseases. Front. Neurosci..

[58] Croese T., Furlan R. (2018). Extracellular vesicles in neurodegenerative diseases. Mol. Asp. Med..

[59] Hill A.F. (2019). Extracellular Vesicles and Neurodegenerative Diseases. J. Neurosci. Off. J. Soc. Neurosci..

[60] You Y., Ikezu T. (2019). Emerging roles of extracellular vesicles in neurodegenerative disorders. Neurobiol. Dis..

[61] Kutchy N.A., Peeples E.S., Sil S., Liao K., Chivero E.T., Hu G., Buch S. (2020). Extracellular Vesicles in Viral Infections of the Nervous System. Viruses.

[62] Saenz-Cuesta M., Osorio-Querejeta I., Otaegui D. (2014). Extracellular Vesicles in Multiple Sclerosis: What are They Telling Us?. Front. Cell. Neurosci..

[63] Osorio-Querejeta I., Alberro A., Munoz-Culla M., Mager I., Otaegui D. (2018). Therapeutic Potential of Extracellular Vesicles for Demyelinating Diseases; Challenges and Opportunities. Front. Mol. Neurosci..

[64] Barreca M.M., Aliotta E., Geraci F. (2017). Extracellular Vesicles in Multiple Sclerosis as Possible Biomarkers: Dream or Reality?. Adv. Exp. Med. Biol..

[65] Quek C., Hill A.F. (2017). The role of extracellular vesicles in neurodegenerative diseases. Biochem. Biophys. Res. Commun..

[66] Vella L.J., Hill A.F., Cheng L. (2016). Focus on Extracellular Vesicles: Exosomes and Their Role in Protein Trafficking and Biomarker Potential in Alzheimer’s and Parkinson’s Disease. Int. J. Mol. Sci..

[67] Bellingham S.A., Guo B.B., Coleman B.M., Hill A.F. (2012). Exosomes: Vehicles for the transfer of toxic proteins associated with neurodegenerative diseases?. Front. Physiol..

[68] Saman S., Kim W., Raya M., Visnick Y., Miro S., Jackson B., McKee A.C., Alvarez V.E., Lee N.C., Hall G.F. (2012). Exosome-associated tau is secreted in tauopathy models and is selectively phosphorylated in cerebrospinal fluid in early Alzheimer disease. J. Biol. Chem..

[69] Rajendran L., Honsho M., Zahn T.R., Keller P., Geiger K.D., Verkade P., Simons K. (2006). Alzheimer’s disease beta-amyloid peptides are released in association with exosomes. Proc. Natl. Acad. Sci. USA.

[70] Emmanouilidou E., Melachroinou K., Roumeliotis T., Garbis S.D., Ntzouni M., Margaritis L.H., Stefanis L., Vekrellis K. (2010). Cell-produced alpha-synuclein is secreted in a calcium-dependent manner by exosomes and impacts neuronal survival. J. Neurosci. Off. J. Soc. Neurosci..

[71] Gomes C., Keller S., Altevogt P., Costa J. (2007). Evidence for secretion of Cu,Zn superoxide dismutase via exosomes from a cell model of amyotrophic lateral sclerosis. Neurosci. Lett..

[72] Grad L.I., Yerbury J.J., Turner B.J., Guest W.C., Pokrishevsky E., O’Neill M.A., Yanai A., Silverman J.M., Zeineddine R., Corcoran L. (2014). Intercellular propagated misfolding of wild-type Cu/Zn superoxide dismutase occurs via exosome-dependent and -independent mechanisms. Proc. Natl. Acad. Sci. USA.

[73] Fevrier B., Vilette D., Archer F., Loew D., Faigle W., Vidal M., Laude H., Raposo G. (2004). Cells release prions in association with exosomes. Proc. Natl. Acad. Sci. USA.

[74] Bakhti M., Winter C., Simons M. (2011). Inhibition of myelin membrane sheath formation by oligodendrocyte-derived exosome-like vesicles. J. Biol. Chem..

[75] Kramer-Albers E.M. (2020). Extracellular vesicles in the oligodendrocyte microenvironment. Neurosci. Lett..

[76] Kramer-Albers E.M., Bretz N., Tenzer S., Winterstein C., Mobius W., Berger H., Nave K.A., Schild H., Trotter J. (2007). Oligodendrocytes secrete exosomes containing major myelin and stress-protective proteins: Trophic support for axons?. Proteom. Clin. Appl..

[77] Frohlich D., Kuo W.P., Fruhbeis C., Sun J.J., Zehendner C.M., Luhmann H.J., Pinto S., Toedling J., Trotter J., Kramer-Albers E.M. (2014). Multifaceted effects of oligodendroglial exosomes on neurons: Impact on neuronal firing rate, signal transduction and gene regulation. Philos. Trans. R. Soc. Lond. Ser. B Biol. Sci..

[78] Kornienko V.N., Pronin I.N., Serkov S., Kornienko V.N., Pronin I.N. (2009). Demyelinating Diseases of the Central Nervous System. Diagnostic Neuroradiology.

[79] Barkhof F., Koeller K.K., Hodler J., Kubik-Huch R.A., von Schulthess G.K. (2020). Demyelinating Diseases of the CNS (Brain and Spine). Diseases of the Brain, Head and Neck, Spine 2020–2023: Diagnostic Imaging.

[80] Alizadeh A., Dyck S.M., Karimi-Abdolrezaee S. (2015). Myelin damage and repair in pathologic CNS: Challenges and prospects. Front. Mol. Neurosci..

[81] Mehndiratta M.M., Gulati N.S. (2014). Central and peripheral demyelination. J. Neurosci. Rural Pract..

[82] van der Knaap M.S., Bugiani M. (2017). Leukodystrophies: A proposed classification system based on pathological changes and pathogenetic mechanisms. Acta Neuropathol..

[83] Love S. (2006). Demyelinating diseases. J. Clin. Pathol..

[84] Filippi M., Bar-Or A., Piehl F., Preziosa P., Solari A., Vukusic S., Rocca M.A. (2018). Multiple sclerosis. Nat. Rev. Dis. Primers.

[85] Storch-Hagenlocher B., Bendszus M., Hähnel S. (2009). Multiple Sclerosis and Other Demyelinating Diseases. Inflammatory Diseases of the Brain.

[86] Grzonka P., Scholz M.C., De Marchis G.M., Tisljar K., Ruegg S., Marsch S., Fladt J., Sutter R. (2020). Acute Hemorrhagic Leukoencephalitis: A Case and Systematic Review of the Literature. Front. Neurol..

[87] Ferren M., Horvat B., Mathieu C. (2019). Measles Encephalitis: Towards New Therapeutics. Viruses.

[88] Rocke Z., Belyayeva M. (2020). Subacute Sclerosing Panencephalitis. StatPearls.

[89] Cowley T.J., Weiss S.R. (2010). Murine coronavirus neuropathogenesis: Determinants of virulence. J. Neurovirol..

[90] Haring J., Perlman S. (2001). Mouse hepatitis virus. Curr. Opin. Microbiol..

[91] Templeton S.P., Perlman S. (2007). Pathogenesis of acute and chronic central nervous system infection with variants of mouse hepatitis virus, strain JHM. Immunol. Res..

[92] O’Gorman C., Lucas R., Taylor B. (2012). Environmental risk factors for multiple sclerosis: A review with a focus on molecular mechanisms. Int. J. Mol. Sci..

[93] Correale J., Gaitan M.I. (2015). Multiple sclerosis and environmental factors: The role of vitamin D, parasites, and Epstein-Barr virus infection. Acta Neurol. Scand..

[94] Calabrese M., Magliozzi R., Ciccarelli O., Geurts J.J., Reynolds R., Martin R. (2015). Exploring the origins of grey matter damage in multiple sclerosis. Nat. Rev. Neurosci..

[95] Cavallo S. (2020). Immune-mediated genesis of multiple sclerosis. J. Transl. Autoimmun..

[96] Lewis P.A., Spillane J.E., Lewis P.A., Spillane J.E. (2019). Multiple Sclerosis. The Molecular and Clinical Pathology of Neurodegenerative Disease.

[97] Virtanen J.O., Jacobson S. (2012). Viruses and multiple sclerosis. Cns Neurol. Disord. Drug Targets.

[98] Donati D. (2020). Viral infections and multiple sclerosis. Drug Discov. Today. Dis. Models.

[99] Tarlinton R.E., Martynova E., Rizvanov A.A., Khaiboullina S., Verma S. (2020). Role of Viruses in the Pathogenesis of Multiple Sclerosis. Viruses.

[100] Derada Troletti C., de Goede P., Kamermans A., de Vries H.E. (2016). Molecular alterations of the blood-brain barrier under inflammatory conditions: The role of endothelial to mesenchymal transition. Biochim. Et Biophys. Acta.

[101] Minagar A., Alexander J.S. (2003). Blood-brain barrier disruption in multiple sclerosis. Mult Scler.

[102] Ulivieri C., Baldari C.T. (2017). Regulation of T Cell Activation and Differentiation by Extracellular Vesicles and Their Pathogenic Role in Systemic Lupus Erythematosus and Multiple Sclerosis. Molecules.

[103] Hagens M., van Berckel B., Barkhof F. (2016). Novel MRI and PET markers of neuroinflammation in multiple sclerosis. Curr. Opin. Neurol..

[104] Tommasin S., Gianni C., De Giglio L., Pantano P. (2019). Neuroimaging Techniques to Assess Inflammation in Multiple Sclerosis. Neuroscience.

[105] Derada Troletti C., Fontijn R.D., Gowing E., Charabati M., van Het Hof B., Didouh I., van der Pol S.M.A., Geerts D., Prat A., van Horssen J. (2019). Inflammation-induced endothelial to mesenchymal transition promotes brain endothelial cell dysfunction and occurs during multiple sclerosis pathophysiology. Cell Death Dis..

[106] Chari D.M. (2007). Remyelination in multiple sclerosis. Int. Rev. Neurobiol..

[107] Hess K., Starost L., Kieran N.W., Thomas C., Vincenten M.C.J., Antel J., Martino G., Huitinga I., Healy L., Kuhlmann T. (2020). Lesion stage-dependent causes for impaired remyelination in MS. Acta Neuropathol..

[108] Carandini T., Colombo F., Finardi A., Casella G., Garzetti L., Verderio C., Furlan R. (2015). Microvesicles: What is the Role in Multiple Sclerosis?. Front. Neurol..

[109] Gyorgy B., Hung M.E., Breakefield X.O., Leonard J.N. (2015). Therapeutic applications of extracellular vesicles: Clinical promise and open questions. Annu. Rev. Pharmacol. Toxicol..

[110] de Jong O.G., Kooijmans S.A.A., Murphy D.E., Jiang L., Evers M.J.W., Sluijter J.P.G., Vader P., Schiffelers R.M. (2019). Drug Delivery with Extracellular Vesicles: From Imagination to Innovation. Acc. Chem. Res..

[111] Murphy D.E., de Jong O.G., Brouwer M., Wood M.J., Lavieu G., Schiffelers R.M., Vader P. (2019). Extracellular vesicle-based therapeutics: Natural versus engineered targeting and trafficking. Exp. Mol. Med..

[112] Xin H., Li Y., Buller B., Katakowski M., Zhang Y., Wang X., Shang X., Zhang Z.G., Chopp M. (2012). Exosome-mediated transfer of miR-133b from multipotent mesenchymal stromal cells to neural cells contributes to neurite outgrowth. Stem Cells.

[113] Zhuang X., Xiang X., Grizzle W., Sun D., Zhang S., Axtell R.C., Ju S., Mu J., Zhang L., Steinman L. (2011). Treatment of brain inflammatory diseases by delivering exosome encapsulated anti-inflammatory drugs from the nasal region to the brain. Mol. Ther. J. Am. Soc. Gene Ther..

[114] Alvarez-Erviti L., Seow Y., Yin H., Betts C., Lakhal S., Wood M.J. (2011). Delivery of siRNA to the mouse brain by systemic injection of targeted exosomes. Nat. Biotechnol..

[115] Scolding N.J., Morgan B.P., Houston W.A., Linington C., Campbell A.K., Compston D.A. (1989). Vesicular removal by oligodendrocytes of membrane attack complexes formed by activated complement. Nature.

[116] Minagar A., Jy W., Jimenez J.J., Sheremata W.A., Mauro L.M., Mao W.W., Horstman L.L., Ahn Y.S. (2001). Elevated plasma endothelial microparticles in multiple sclerosis. Neurology.

[117] Jy W., Minagar A., Jimenez J.J., Sheremata W.A., Mauro L.M., Horstman L.L., Bidot C., Ahn Y.S. (2004). Endothelial microparticles (EMP) bind and activate monocytes: Elevated EMP-monocyte conjugates in multiple sclerosis. Front. Biosci. A J. Virtual Libr..

[118] Jimenez J., Jy W., Mauro L.M., Horstman L.L., Ahn E.R., Ahn Y.S., Minagar A. (2005). Elevated endothelial microparticle-monocyte complexes induced by multiple sclerosis plasma and the inhibitory effects of interferon-beta 1b on release of endothelial microparticles, formation and transendothelial migration of monocyte-endothelial microparticle complexes. Mult Scler.

[119] Wimmer I., Tietz S., Nishihara H., Deutsch U., Sallusto F., Gosselet F., Lyck R., Muller W.A., Lassmann H., Engelhardt B. (2019). PECAM-1 Stabilizes Blood-Brain Barrier Integrity and Favors Paracellular T-Cell Diapedesis Across the Blood-Brain Barrier During Neuroinflammation. Front. Immunol..

[120] Losy J., Niezgoda A., Wender M. (1999). Increased serum levels of soluble PECAM-1 in multiple sclerosis patients with brain gadolinium-enhancing lesions. J. Neuroimmunol..

[121] Kuenz B., Lutterotti A., Khalil M., Ehling R., Gneiss C., Deisenhammer F., Reindl M., Berger T. (2005). Plasma levels of soluble adhesion molecules sPECAM-1, sP-selectin and sE-selectin are associated with relapsing-remitting disease course of multiple sclerosis. J. Neuroimmunol..

[122] Niezgoda A., Losy J. (2002). Pecam-1 expression in patients with relapsing-remitting multiple sclerosis. Folia Morphol..

[123] Verderio C., Muzio L., Turola E., Bergami A., Novellino L., Ruffini F., Riganti L., Corradini I., Francolini M., Garzetti L. (2012). Myeloid microvesicles are a marker and therapeutic target for neuroinflammation. Ann. Neurol..

[124] Marcos-Ramiro B., Oliva Nacarino P., Serrano-Pertierra E., Blanco-Gelaz M.A., Weksler B.B., Romero I.A., Couraud P.O., Tunon A., Lopez-Larrea C., Millan J. (2014). Microparticles in multiple sclerosis and clinically isolated syndrome: Effect on endothelial barrier function. BMC Neurosci..

[125] O’Brien K., Breyne K., Ughetto S., Laurent L.C., Breakefield X.O. (2020). RNA delivery by extracellular vesicles in mammalian cells and its applications. Nat. Rev. Mol. Cell Biol..

[126] Ebrahimkhani S., Vafaee F., Young P.E., Hur S.S.J., Hawke S., Devenney E., Beadnall H., Barnett M.H., Suter C.M., Buckland M.E. (2017). Exosomal microRNA signatures in multiple sclerosis reflect disease status. Sci. Rep..

[127] Selmaj I., Cichalewska M., Namiecinska M., Galazka G., Horzelski W., Selmaj K.W., Mycko M.P. (2017). Global exosome transcriptome profiling reveals biomarkers for multiple sclerosis. Ann. Neurol..

[128] Chen C., Zhou Y., Wang J., Yan Y., Peng L., Qiu W. (2018). Dysregulated MicroRNA Involvement in Multiple Sclerosis by Induction of T Helper 17 Cell Differentiation. Front. Immunol..

[129] Jagot F., Davoust N. (2016). Is It worth Considering Circulating microRNAs in Multiple Sclerosis?. Front. Immunol..

[130] Dolati S., Marofi F., Babaloo Z., Aghebati-Maleki L., Roshangar L., Ahmadi M., Rikhtegar R., Yousefi M. (2018). Dysregulated Network of miRNAs Involved in the Pathogenesis of Multiple Sclerosis. Biomed. Pharmacother..

[131] Junker A., Krumbholz M., Eisele S., Mohan H., Augstein F., Bittner R., Lassmann H., Wekerle H., Hohlfeld R., Meinl E. (2009). MicroRNA profiling of multiple sclerosis lesions identifies modulators of the regulatory protein CD47. Brain A J. Neurol..

[132] Zhang J., Cheng Y., Cui W., Li M., Li B., Guo L. (2014). MicroRNA-155 modulates Th1 and Th17 cell differentiation and is associated with multiple sclerosis and experimental autoimmune encephalomyelitis. J. Neuroimmunol..

[133] Lennon V.A., Kryzer T.J., Pittock S.J., Verkman A.S., Hinson S.R. (2005). IgG marker of optic-spinal multiple sclerosis binds to the aquaporin-4 water channel. J. Exp. Med..

[134] Lee J., McKinney K.Q., Pavlopoulos A.J., Han M.H., Kim S.H., Kim H.J., Hwang S. (2016). Exosomal proteome analysis of cerebrospinal fluid detects biosignatures of neuromyelitis optica and multiple sclerosis. Clin. Chim. Acta Int. J. Clin. Chem..

[135] Bejerot S., Hesselmark E., Mobarrez F., Wallen H., Hietala M.A., Nybom R., Wetterberg L. (2019). Neuromyelitis optica spectrum disorder with increased aquaporin-4 microparticles prior to autoantibodies in cerebrospinal fluid: A case report. J. Med Case Rep..

[136] Adiele R., Adiele C. (2014). Progressive Multifocal Leukoencephalopathy. J. Mult Scler..

[137] Padgett B.L., Walker D.L., ZuRhein G.M., Eckroade R.J., Dessel B.H. (1971). Cultivation of papova-like virus from human brain with progressive multifocal leucoencephalopathy. Lancet.

[138] Santiana M., Altan-Bonnet N. (2019). Insane in the Membrane: Glial Extracellular Vesicles Transmit Polyomaviruses. mBio.

[139] Harypursat V., Zhou Y., Tang S., Chen Y. (2020). JC Polyomavirus, progressive multifocal leukoencephalopathy and immune reconstitution inflammatory syndrome: A review. Aids Res. Ther..

[140] Mills E.A., Mao-Draayer Y. (2018). Understanding Progressive Multifocal Leukoencephalopathy Risk in Multiple Sclerosis Patients Treated with Immunomodulatory Therapies: A Bird’s Eye View. Front. Immunol..

[141] Neu U., Maginnis M.S., Palma A.S., Stroh L.J., Nelson C.D., Feizi T., Atwood W.J., Stehle T. (2010). Structure-function analysis of the human JC polyomavirus establishes the LSTc pentasaccharide as a functional receptor motif. Cell Host Microbe.

[142] Assetta B., Maginnis M.S., Gracia Ahufinger I., Haley S.A., Gee G.V., Nelson C.D., O’Hara B.A., Allen Ramdial S.A., Atwood W.J. (2013). 5-HT2 receptors facilitate JC polyomavirus entry. J. Virol..

[143] Morris-Love J., Gee G.V., O’Hara B.A., Assetta B., Atkinson A.L., Dugan A.S., Haley S.A., Atwood W.J. (2019). JC Polyomavirus Uses Extracellular Vesicles To Infect Target Cells. mBio.

[144] O’Hara B.A., Morris-Love J., Gee G.V., Haley S.A., Atwood W.J. (2020). JC Virus infected choroid plexus epithelial cells produce extracellular vesicles that infect glial cells independently of the virus attachment receptor. Plos Pathog..

[145] Giraudon P., Bernard A. (2009). Chronic viral infections of the central nervous system: Aspects specific to multiple sclerosis. Rev. Neurol..

[146] Simmons A. (2001). Herpesvirus and multiple sclerosis. Herpes J. IHMF.

[147] Bello-Morales R., Andreu S., Lopez-Guerrero J.A. (2020). The Role of Herpes Simplex Virus Type 1 Infection in Demyelination of the Central Nervous System. Int. J. Mol. Sci..

[148] Santiago O., Gutierrez J., Sorlozano A., de Dios Luna J., Villegas E., Fernandez O. (2010). Relation between Epstein-Barr virus and multiple sclerosis: Analytic study of scientific production. Eur. J. Clin. Microbiol. Infect. Dis. Off. Publ. Eur. Soc. Clin. Microbiol..

[149] Bar-Or A., Pender M.P., Khanna R., Steinman L., Hartung H.-P., Maniar T., Croze E., Aftab B.T., Giovannoni G., Joshi M.A. (2020). Epstein–Barr Virus in Multiple Sclerosis: Theory and Emerging Immunotherapies. Trends Mol. Med..

[150] Pormohammad A., Azimi T., Falah F., Faghihloo E. (2018). Relationship of human herpes virus 6 and multiple sclerosis: A systematic review and meta-analysis. J. Cell. Physiol..

[151] Fotheringham J., Jacobson S. (2005). Human herpesvirus 6 and multiple sclerosis: Potential mechanisms for virus-induced disease. Herpes J. IHMF.

[152] Marrodan M., Alessandro L., Farez M.F., Correale J. (2019). The role of infections in multiple sclerosis. Mult Scler.

[153] Voumvourakis K.I., Kitsos D.K., Tsiodras S., Petrikkos G., Stamboulis E. (2010). Human herpesvirus 6 infection as a trigger of multiple sclerosis. Mayo Clin. Proc..

[154] Guan Y., Jakimovski D., Ramanathan M., Weinstock-Guttman B., Zivadinov R. (2019). The role of Epstein-Barr virus in multiple sclerosis: From molecular pathophysiology to in vivo imaging. Neural Regen. Res..

[155] Virtanen J.O., Wohler J., Fenton K., Reich D.S., Jacobson S. (2014). Oligoclonal bands in multiple sclerosis reactive against two herpesviruses and association with magnetic resonance imaging findings. Mult. Scler..

[156] Rostrom B., Link H., Laurenzi M.A., Kam-Hansen S., Norrby E., Wahren B. (1981). Viral antibody activity of oligoclonal and polyclonal immunoglobulins synthesized within the central nervous system in multiple sclerosis. Ann. Neurol..

[157] Virtanen J.O., Pietilainen-Nicklen J., Uotila L., Farkkila M., Vaheri A., Koskiniemi M. (2011). Intrathecal human herpesvirus 6 antibodies in multiple sclerosis and other demyelinating diseases presenting as oligoclonal bands in cerebrospinal fluid. J. Neuroimmunol..

[158] Bello-Morales R., Ripa I., Lopez-Guerrero J.A. (2020). Extracellular Vesicles in Viral Spread and Antiviral Response. Viruses.

[159] Kalamvoki M., Du T., Roizman B. (2014). Cells infected with herpes simplex virus 1 export to uninfected cells exosomes containing STING, viral mRNAs, and microRNAs. Proc. Natl. Acad. Sci. USA.

[160] Kalamvoki M., Deschamps T. (2016). Extracellular vesicles during Herpes Simplex Virus type 1 infection: An inquire. Virol. J..

[161] Bello-Morales R., Praena B., de la Nuez C., Rejas M.T., Guerra M., Galan-Ganga M., Izquierdo M., Calvo V., Krummenacher C., Lopez-Guerrero J.A. (2018). Role of Microvesicles in the Spread of Herpes Simplex Virus 1 in Oligodendrocytic Cells. J. Virol..

[162] Morandi E., Tanasescu R., Tarlinton R.E., Constantinescu C.S., Zhang W., Tench C., Gran B. (2017). The association between human endogenous retroviruses and multiple sclerosis: A systematic review and meta-analysis. PLoS ONE.

[163] Christensen T. (2005). Association of human endogenous retroviruses with multiple sclerosis and possible interactions with herpes viruses. Rev. Med Virol..

[164] Christensen T. (2017). Human endogenous retroviruses in the aetiology of MS. Acta Neurol. Scand..

[165] Alvarez-Lafuente R., Garcia-Montojo M., De Las Heras V., Dominguez-Mozo M.I., Bartolome M., Benito-Martin M.S., Arroyo R. (2008). Herpesviruses and human endogenous retroviral sequences in the cerebrospinal fluid of multiple sclerosis patients. Mult. Scler..

[166] Kriesel J.D., Bhetariya P.J., Chan B.K., Wilson T., Fischer K.F. (2017). Enrichment of Retroviral Sequences in Brain Tissue from Patients with Severe Demyelinating Diseases. J. Emerg. Dis. Virol..

[167] Perron H., Garson J.A., Bedin F., Beseme F., Paranhos-Baccala G., Komurian-Pradel F., Mallet F., Tuke P.W., Voisset C., Blond J.L. (1997). Molecular identification of a novel retrovirus repeatedly isolated from patients with multiple sclerosis. The Collaborative Research Group on Multiple Sclerosis. Proc. Natl. Acad. Sci. USA.

[168] Sotgiu S., Mameli G., Serra C., Zarbo I.R., Arru G., Dolei A. (2010). Multiple sclerosis-associated retrovirus and progressive disability of multiple sclerosis. Mult. Scler..

[169] Johnson W.E. (2019). Origins and evolutionary consequences of ancient endogenous retroviruses. Nat. Rev. Microbiol..

[170] Mi S., Lee X., Li X., Veldman G.M., Finnerty H., Racie L., LaVallie E., Tang X.Y., Edouard P., Howes S. (2000). Syncytin is a captive retroviral envelope protein involved in human placental morphogenesis. Nature.

[171] Wang X., Huang J., Zhu F. (2018). Human Endogenous Retroviral Envelope Protein Syncytin-1 and Inflammatory Abnormalities in Neuropsychological Diseases. Front. Psychiatry.

[172] Kremer D., Schichel T., Forster M., Tzekova N., Bernard C., van der Valk P., van Horssen J., Hartung H.P., Perron H., Kury P. (2013). Human endogenous retrovirus type W envelope protein inhibits oligodendroglial precursor cell differentiation. Ann. Neurol..

[173] Ruprecht K., Obojes K., Wengel V., Gronen F., Kim K.S., Perron H., Schneider-Schaulies J., Rieckmann P. (2006). Regulation of human endogenous retrovirus W protein expression by herpes simplex virus type 1: Implications for multiple sclerosis. J. Neurovirol..

[174] Uygur B., Melikov K., Arakelyan A., Margolis L.B., Chernomordik L.V. (2019). Syncytin 1 dependent horizontal transfer of marker genes from retrovirally transduced cells. Sci. Rep..

[175] Vargas A., Zhou S., Ethier-Chiasson M., Flipo D., Lafond J., Gilbert C., Barbeau B. (2014). Syncytin proteins incorporated in placenta exosomes are important for cell uptake and show variation in abundance in serum exosomes from patients with preeclampsia. FASEB J. Off. Publ. Fed. Am. Soc. Exp. Biol..

